# Placental crises: disruptive selection and maternal under‐investment as the foundations of mammalian placental evolution and dysfunction

**DOI:** 10.1002/brv.70139

**Published:** 2026-01-31

**Authors:** Davis Laundon, Neil J. Gostling, Ian G. Reddin, Bram G. Sengers, Pascale Chavatte‐Palmer, Rohan M. Lewis

**Affiliations:** ^1^ School of Human Development and Health, Faculty of Medicine, IDS Building University of Southampton, Southampton General Hospital Southampton SO16 6YD UK; ^2^ Institute for Life Sciences, University of Southampton University Rd, Highfield Southampton SO17 1BJ UK; ^3^ School of Biological Sciences, Faculty of Environmental and Life Sciences University of Southampton University Rd, Highfield Southampton SO17 1BJ UK; ^4^ School of Cancer Sciences, Faculty of Medicine, IDS Building University of Southampton Southampton SO16 6YD UK; ^5^ Bio‐R Bioinformatics Research Facility, Faculty of Medicine, IDS Building University of Southampton Southampton SO16 6YD UK; ^6^ School of Engineering, Faculty of Engineering and Physical Sciences University of Southampton University Road Southampton SO17 1BJ UK; ^7^ Université Paris‐Saclay, UVSQ, INRAE, BREED Jouy‐en‐Josas 78350 France; ^8^ Ecole Nationale Vétérinaire d’Alfort, BREED Maisons‐Alfort 94700 France

**Keywords:** placenta, mammal, disruptive selection, reproductive dysfunction, maternal investment

## Abstract

Among the vertebrates, mammals are notable for the dominance of live birth and placental nutrition. The structural diversity of the mammalian placenta is remarkable, despite sharing a single common ancestor and conserved physiological functions. Historically, investigations into the evolution of the mammalian placenta have been grounded in ‘the efficiency paradigm’, i.e. the assumption that certain placental configurations permit easier nutrient exchange, but this paradigm has struggled to explain the diversity of mammalian placentation strategies. Here, we propose a new paradigm to understand mammalian placental evolution. Using multidimensional plotting of recorded placental structures, quantitative metrics for mammalian maternal investment, and illustrative computational modelling of physiological processes, we argue that the ancestral mammalian placenta is not a streamlined ‘highly efficient’ design, but rather a product of low maternal investment, with fitness costs that manifest as gestational demand increases. Expansion of small mammals into larger‐bodied, longer‐lived niches induces a ‘placental crisis’ characterised by maternal under‐investment and chronic gestational dysfunction, triggering an arms race through the interaction of disruptive selection and materno‐fetal conflict. We propose the acute severity of the placental crisis is the foundation of placental evolution. We go on to argue that some primates are currently in a state of placental crisis and that maternal under‐investment and inappropriate placentation are the evolutionary foundations of human gestational dysfunctions such as pre‐eclampsia. We conclude that the ancestral mammalian placenta was not an efficiently optimised design that allowed placentation to dominate the clade, but rather an idiosyncrasy of mammal‐specific biology, which likely hindered mammalian expansion into larger‐bodied niches.

## I. INTRODUCTION

### (1) Maternal investment and reproductive strategies vary widely across vertebrates

**Fig. 1:**
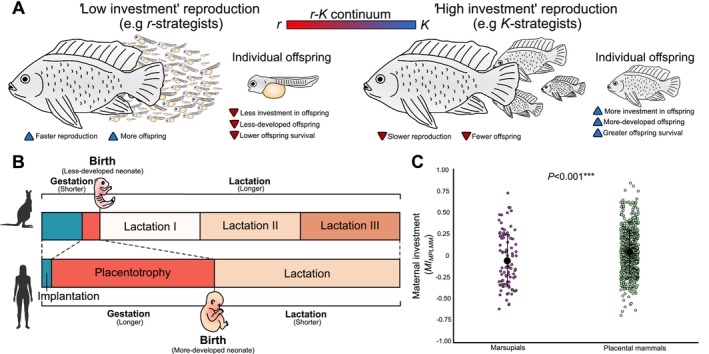
Vertebrates exhibit wide variations in reproductive strategy and maternal investment between groups. (A) Diagrammatic summary of trade‐offs in reproductive strategy between *r‐*selected and *K*‐selected vertebrates. (B) Differential partitioning of relative nutrient‐provisioning strategies between marsupials and placental mammals. Adapted from Abbot & Capra (2017). (C) As a group, marsupials invest less in reproduction than placental mammals as quantified by maternal investment from a multiple phylogenetic linear mixed model (MI_MPLMM_) (Huijsmans *et al*., 2024). Error bars are mean ± standard deviation (SD).

There exists a remarkable diversity of reproductive strategies across the vertebrates (Mayr, 1963; Angelini & Ghiara, 1984; Crespi & Semeniuk, 2004; Famoso, Hopkins, & Davis, 2018). A key difference is the level of resources parentally invested in any given offspring, which disproportionately come from the mother (Trivers, 2017). A well‐described example of this phenomenon is the *r*–*K* continuum of reproductive strategies (Fig. 1A) (Pianka, 1970; MacArthur & Wilson, 2001). This spectrum ranges from species that have many offspring at a time with low per‐offspring parental investment (*r*‐strategy) to those that produce few offspring with a high parental investment in each individual offspring (*K*‐strategy). The terms *r* and *K* are from population dynamics equations, with *r* being rate of population growth and *K* the carrying capacity of the environment (Pianka, 1970). As a general rule, the *r*‐strategy is associated with changeable environments and fluctuating population sizes while the *K*‐strategy is associated with stable environments and population sizes, where individuals must compete for limited resources. Among placental mammals, species occupy different positions along this continuum. For example, mice align more closely with *r*‐strategies, while elephants exemplify *K*‐strategies. Towards the *r*‐ end, mothers produce many small, underdeveloped (‘altricial’) offspring with low individual survival rates, while at the *K*‐ end, they produce fewer, larger, well‐developed (‘precocial’) offspring with higher survival rates. Although there is an inverse correlation between offspring size and litter number (Lack, 1967), this does not necessarily equate to equal total parental investment across the *r*–*K* continuum. Assuming evolutionary fitness is an organism's ability to reproduce *via* offspring that reproduce themselves (Fisher, 1930; Jost, 2003), then individual *r‐*selected offspring are less capable of fulfilling this objective and are less fit than their *K*‐selected counterparts. Given the much larger numbers of *r*‐selected offspring, it is tempting to assume that, overall, *r*–*K* continuum strategies contribute equal resources to reproduction and vary only in response to external ecobiological variables (Pianka, 1970). However, organisms must also trade off resource investment in somatic maintenance and increased investment in reproduction induces reduced somatic growth (Reznick, 1983; Auer *et al*., 2010), lifespan (Smith, 1958; Cox *et al*., 2010; Flatt, 2011), and innate immunity (Adamo, Jensen & Younger, 2001; Neggazi *et al*., 2016; Schwenke, Lazzaro & Wolfner, 2016). We cannot therefore assume that all groups, given their unique metabolic demands and ecological niches, can invest an equal share of energy budgets into reproduction. Under‐investment in reproduction could constitute a ‘cost‐cutting’ strategy.

Mammals exhibit wide variation in reproductive strategies (Weir & Rowlands, 1973; Derrickson, 1992), and offspring can also be considered along the *r*–*K* continuum, or simply by their development at birth along the altricial–precocial spectrum (Portmann, 1939; Derrickson, 1992; Scheiber *et al*., 2017; Augustine, Lika & Kooijman, 2019). An extreme example of differential investment strategies occurs between marsupials and placental mammals (Fig. 1B). Marsupials do receive gestational nutrients *via* a placenta, however, as a group, gestation is shorter relative to lactation than in placental mammals and neonates are born in a highly altricial state (Renfree, 2010; Abbot & Capra, 2017). Nutrient provisioning in marsupials is dominated by postpartum lactation. Again, it has been assumed that these differences are merely matters of nutrient partitioning and that overall marsupials achieve a similar (Hayssen, Lacy & Parker, 1985) or even higher (Thompson & Nicoll, 1986) level of maternal investment than placental mammals. It has been historically difficult to compare relative levels of maternal investment across distantly related mammal groups (Gittleman & Thompson, 1988). Recently, a holistic maternal investment metric (MI_MPLMM_) (maternal investment from a multiple phylogenetic linear mixed model) was developed from the allometric scaling of 738 mammal species, providing a quantitative metric inclusive of variables such as litter size, offspring mass at weaning, adult body size, and investment duration (Huijsmans *et al*., 2024). Comparison of this metric between mammalian subclasses shows that, as a group, placental mammals invest significantly more overall in reproduction than marsupials (Fig. 1C) (Huijsmans *et al*., 2024), supporting the conclusions of some smaller scale experimental studies (Hsu, Garton & Harder, 1999).

### (2) Viviparity and placentation evolved only once in mammals and came to dominate the group

**Fig. 2:**
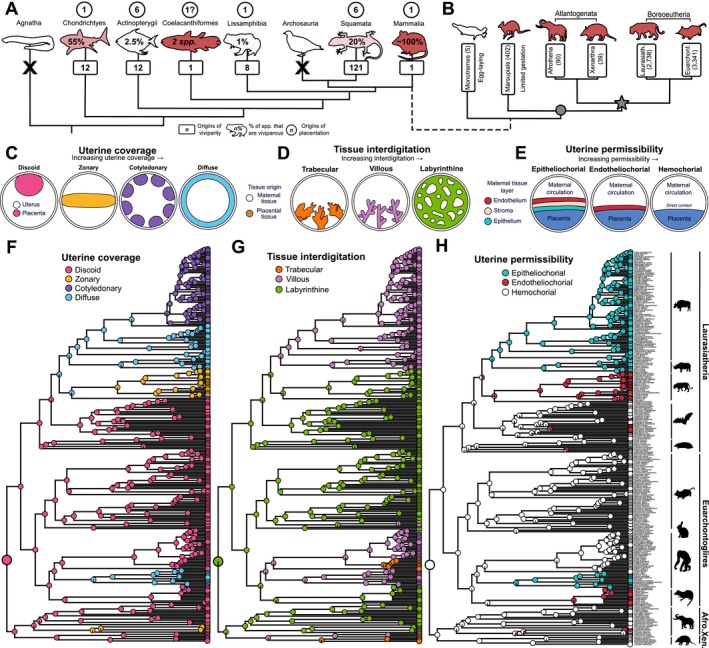
Despite sharing a single evolutionary origin, the mammalian placenta is remarkably structurally diverse. (A) Phylogenetic visualisation of the number of convergent origins of viviparity (squares), complex nutritive placentation (circles), and percentage of viviparous species (% inside silhouette) across major vertebrate groups. Silhouettes are coloured by % of viviparous species. Data from Whittington *et al*. (2024). (B) Mammalian viviparity evolved once (grey‐filled circle) and chorioallantoic placentation (star) emerged as the dominant form of nutrient provisioning in the group. Laurasiath. = Laurasiatheria, Euarchont. = Euarchontoglires. Species data from Burgin *et al*. (2018). (C–E) Diagrammatic summary of so‐called ‘placental type’ categories across structural scales: uterine coverage (C), tissue interdigitation (D), and uterine permissibility (E). Schema adapted from Laundon *et al*. (2023*a*), Furukawa *et al*. (2014) and Carter (2024). (F–H) Phylogenetic distribution and ancestral trait reconstruction of placental type categories C–E for each major structural scale across the Placentalia. Data from Elliot & Crespi (2009).

Viviparity (live birth) has arisen over 150 times independently in vertebrates from egg‐laying ancestors (Fig. 2A; see online Supporting Information, Appendix S1, for methodological details used in the creation of the figures included in this review, and Data S1 for the data sets analysed) (Amoroso, 1959; Blackburn, 1992, 2015; Whittington *et al*., 2022*a*; Whittington, Hodgson, & Friesen 2024; Li *et al*., 2025). Viviparity allows greater maternal control of nutrient allocation, niche exploration, and protection of offspring from predation (Whittington *et al*., 2022*a*), at the cost of reduced maternal mobility, increased resource demands, and increased materno‐fetal conflict (Zeh & Zeh, 2000; Pollux *et al*., 2014; Whittington *et al*., 2022*b*; Morrison *et al*., 2024). The evolution of viviparity may also be coupled to innovation in nutrient provisioning, often from lecithotrophy (a finite deposited yolk source) to matrotrophy (continuous nutrient provisioning from the mother) (Blackburn, 1992; Ostrovsky *et al*., 2016; Whittington *et al*., 2022*b*). The most complex form of matrotrophy is placentotrophy, where nutrients flow from mother to fetus *via* a fetally derived placenta which mediates physiological exchange (Mossman, 1937; Burton, 2022). Complex nutritive placentas have originated ≥16 times independently in viviparous vertebrates, often from diverse precursory tissues (Whittington *et al*., 2024).

Placentotrophy dominates nutrient provisioning in the mammals to an extent unparalleled among vertebrates, with each of the ~6,500 species placentotrophic to some degree (Fig. 2B) (Freyer, Zeller & Renfree, 2003; Ferner, Schultz & Zeller, 2017; Mika *et al*., 2022). All mammals except monotremes bear live young and ~94% of mammal species belong to the Placentalia [the ‘placental mammals’, so‐called because the bulk of nutrient provisioning comes from a definitive chorioallantoic placenta (Fleming, 1822; Owen, 1837; Gill, 1874; Rougier, Wible & Novacek, 1998; Archibald, Averianov & Ekdale, 2001; Burgin *et al*., 2018)]. Viviparity and placentation evolved only once in the mammals, and all mammalian placentas originate from a common ancestor ~130 Ma (Haeckel, 1868; Wildman *et al*., 2006; Griffith & Wagner, 2017). However, despite sharing a single evolutionary origin and conserved functions, the placenta is arguably the most structurally diverse mammalian organ (Amoroso, 1959; Enders, 1965; Wildman *et al*., 2006; Furukawa, Kuroda & Sugiyama, 2014; Carter, 2018; Laundon *et al*., 2023*a*). The reasons for this striking structural diversity are poorly understood. The radical structural diversity of the mammalian placenta is observed across scales and is described using a categorical schema (Fig. 2C–E) (reviewed in detail in Mayr, 1963; Furukawa *et al*., 2014; Burton, 2022; Laundon *et al*., 2023*a*; Baker, 2024). Placentas can be ranked by the relative amount of uterus they cover (Fig. 2C), the degree of interdigitation with maternal tissue (Fig. 2D), and the number of tissue layers separating the placenta from maternal circulation (i.e. how permissible the uterus is to placental ‘invasion’; Fig. 2E) (Mayr, 1963; Furukawa *et al*., 2014; Burton, 2022; Laundon *et al*., 2023*a*; Baker, 2024).

## II. THE ‘EFFICIENCY PARADIGM’ OF MAMMALIAN PLACENTAL EVOLUTION DOES NOT WORK

**Fig. 3:**
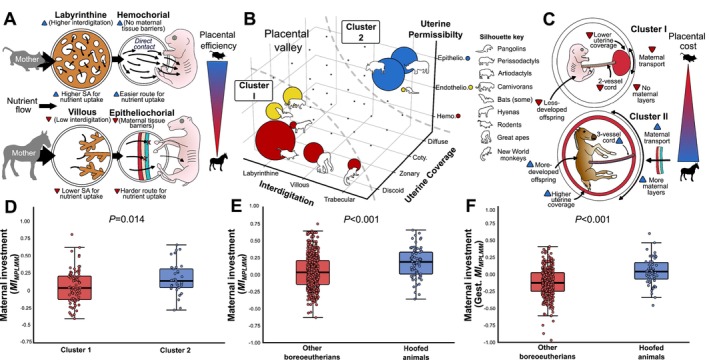
(A) Diagrammatic representation of the current dominant ‘efficiency paradigm’ suggesting that, all things being equal, more interdigitated placentas in direct contact with maternal blood permit more efficient nutrient exchange. (B) Three‐dimensional distribution of structural combinations from recorded extant mammals (Elliot & Crespi, 2009) shows two distinct clusters. Dot size corresponds to abundance of structural combination. Asterisks represent possible combinations represented by no known mammal. Silhouettes (key on right) show major representative example mammal groups that display each placental type. Coty., cotyledonary; Epithelio., epitheliochorial; Endothelio., endotheliochorial; Hemo. hemochorial. (C) Diagrammatic representation of the ‘placental cost’ paradigm advanced in this review, highlighting key aspects of pregnancies with Cluster 2 placentas that require greater resource allocation. (D–F) Comparison of maternal investment as quantified by maternal investment from a multiple phylogenetic linear mixed model (*MI*_*MPLMM*_; Huijsmans *et al*., 2024) between placenta types. (D) Species with Cluster 2 placentas (as plotted in B) invest significantly more in reproduction than Cluster 1 placentas. This is also true when hoofed animals (perissodactyls + artiodactyls) are compared to other boreoeutherians for the entire maternal investment (E), or when only the gestational (Gest.) contribution to reproduction is considered (F).

The evolutionary drivers and selection mechanisms underpinning the vast diversification of mammalian placentas have proved difficult to resolve. Central to previous investigations is the ‘efficiency paradigm’ (a term we coin here), which states that more intimately interdigitated placentas with more direct contact with maternal blood permit a higher efficiency of nutrient transfer from mother to fetus due to a higher relative surface area for nutrient uptake and fewer cellular barriers between fetal and maternal circulation (Fig. 3A) (Huxley, 1880; Strahl, 1906; Grosser, 1909, 1933; Leiser & Kaufmann, 1994; Laundon *et al*., 2023*a*). This paradigm fits the historic hypothesis that the ancestral placenta of placental mammals was epitheliochorial (only in contact with the uterine epithelium) and that placental evolution flowed in the direction of increased efficiency (Turner, 1876; Huxley, 1880). However subsequent ancestral trait reconstructions (Wildman *et al*., 2006; Elliot & Crespi, 2009; Mika *et al*., 2022), including our own (Fig. 2F–H), have indicated that the ancestral placenta of placental mammals was discoid, labyrinthine, and hemochorial (directly in contact with maternal blood), and thus highly ‘efficient’. Such placentas are thought to permit higher fetal growth rates (Leiser & Kaufmann, 1994; Capellini, Venditti & Barton, 2011; Lewitus & Soligo, 2011) and higher fetal brain development (Elliot & Crespi, 2008), although this has been contested (Martin, 2008). Why then, evolve a derived ‘low‐efficiency’ placenta?

Proposed explanations for the emergence of epitheliochorial placentation centre on the phenomenon of ‘materno‐fetal conflict’ (Moore & Haig, 1991; Haig, 1993; Klisch & Mess, 2007; Wang *et al*., 2013; Kazemian *et al*., 2019; Laundon *et al*., 2024*c*). As the fetus is half paternal and half maternal, the fitness interests of the mother and fetus can become misaligned (Haig, 1993), especially for polygynous species (>90% of all mammal species) (Clutton‐Brock, 1989). In this scenario, a fetus may extract excessive nutrients from the mother, harming her future reproductive output, while benefitting paternal fitness. It is therefore proposed that the evolution of ‘less‐efficient’ nutrient transfer evolved in species with large offspring, so the mother may withhold nutrients for her future reproduction in a phenomenon called ‘maternal constraint’ (Gluckman *et al*., 1992; Allen *et al*., 2002; Lewis, Cleal & Hanson, 2012). Other competing factors may also select against placental efficiency, such as reducing vertical pathogen transmission (Capellini, Nunn & Barton, 2015), immunorejection of the fetus (Moffett & Loke, 2006; Hemberger, 2013; Roberts, Green & Schulz, 2016), and damage to the mother by reactive oxygen species (Elliot, 2016), although this is far from clear. Under any of these proposed scenarios, the efficiency paradigm is most widely accepted as central to the interpretation.

We argue that the efficiency paradigm cannot explain mammalian placental evolution and diversification. We do not challenge the central premise that, all other things being equal, more intimate materno‐fetal tissue interdigitation and fewer cellular barriers between maternal and fetal circulations should permit easier nutrient transfer. However, all things are not equal. Plotting of the distribution of placental type combinations for mammal species (Elliot & Crespi, 2009) in a three‐dimensional morphospace shows the grouping of structural combinations into two distinct clusters separated by a ‘placental valley’ of unpopulated hypothetical placental configurations (Fig. 3B). Indeed, only 25% of the 36 possible combinations are occupied, with 72% of recorded mammal species found at just three points located at opposite poles of the placental morphospace, suggestive of disruptive selection (Smith, 1962).

Co‐occurrence of placental types may compensate for efficiency limitations at lower scales, such as placentas with lower interdigitation/uterine permissibility covering a larger area of the uterus (Leiser & Kaufmann, 1994; Burton, 2022; Laundon *et al*., 2023*a*), but this is rarely considered in investigations grounded in the efficiency paradigm. Likewise, there is a paradox at the heart of the efficiency paradigm, namely that highly ‘efficient’ hemochorial labyrinthine placentas typically produce small, altricial offspring (Capellini *et al*., 2011; Garratt *et al*., 2013) (Fig. 3C). Why would high‐efficiency placentas produce offspring with lower survival prospects, motility, birth mass, and trophic independence? In addition to a less‐developed offspring and lower uterine coverage, some of the most *r‐*selected species with hemochorial labyrinthine placentas such as rats and mice also have umbilical cords which have lost a vessel, which our computational modelling suggests confers reduced exchange flux relative to larger‐bodied animals with additional vessels (Wan *et al*., 2025). We should not view the placenta in isolation but rather as part of an integrated ‘fetoplacental unit’ inclusive of fetus and cord, in relation to maternal adaptations. The absence of maternal tissue layers and nutrient transport will require less maternal investment to maintain than in epitheliochorial placentas. Taken together, we argue that the hemochorial labyrinthine unit does not appear as a streamlined organisation to maximise nutrient acquisition but rather represents an assemblage with low individual investment from the mother.

## III. THE PLACENTAL COST HYPOTHESIS: AN INVERTED PARADIGM

We argue that resolving the mechanisms and drivers for the evolution of the mammalian placenta has proved challenging because the efficiency paradigm does not fit our understanding of mammalian reproductive evolution. We instead propose that the ancestral design represents a low‐maternal‐investment strategy, resulting in design flaws, that is only adaptive in highly *r*‐selected mammals and is increasingly maladaptive over longer gestation periods or for larger offspring. We suggest that the evolution of longer‐lived, larger‐bodied, *K*‐selected mammal groups induced a reproductive crisis as this organisation began to fail and, through the dynamic interaction of disruptive selection and materno‐fetal conflict, drove placental reorganisation and increased maternal investment into the pregnancy (fetoplacental unit + maternal adaptations) to support these gestationally demanding offspring.

We can initially test the hypothesis that Cluster 1 placentas (Fig. 3B) are a product of lower maternal investment using the maternal investment metric (MI_MPLMM_) (Huijsmans *et al*., 2024). When we integrate previous data on recorded mammalian placental types (Elliot & Crespi, 2009) with MI_MPLMM_ life‐history data from Huijsmans *et al*. (2024), we can directly compare the relative amount of total maternal investment between mammals with Cluster 1 and Cluster 2 placentas (Fig. 3D–F). As our hypothesis predicts, Cluster 2 mammals invest significantly more (*P* = 0.014) in reproduction overall than Cluster 1 mammals (Fig. 3D). The number of recorded placental configurations for mammal species is much lower (*N* = 290) than the number for which MI_MPLMM_ values are available (*N* = 736), and recorded entries often select for outliers so, although still a clear difference, the distribution presented in Fig. 3D is probably noisier than the biological reality. Comparing ‘hoofed animals’ (perissodactyls + artiodactyls) with all other boreoeutherians, hoofed animals as a group invest significantly more (*P* < 0.001) in reproduction (Fig. 3E). Recalculating *MI*_*MPLMM*_ for only the gestational portion of reproductive investment (Gest. MI_MPLMM_; see Appendix S1) results in an even stronger difference (*P* < 0.001) (Fig. 3F).

**Fig. 4:**
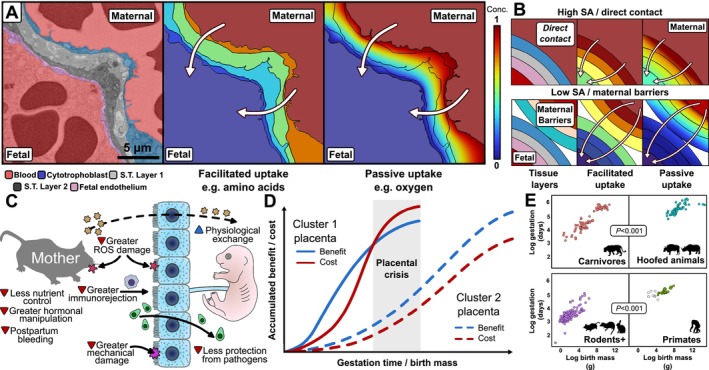
The ancestral placenta develops physiological costs when scaled up that do not manifest under low gestational demand. (A) Illustrative modelling of diffusive processes in an E18.5 mouse placenta. Colours in the two panels on the right represent relative nutrient concentration (see scale bar). S.T. = syncytiotrophoblast. (B) Diagrammatic visualisation of how both forms of nutrient uptake from A will be limited by additional maternal tissue barriers. SA, surface area. (C) Although a Cluster 1‐type placenta (see Fig. 3B) may have easier physiological nutrient exchange, this same design also confers many costs. ROS, reactive oxygen species. (D) The costs of Cluster 1 placentas will manifest as birth mass and gestation length increase, causing a ‘placental crisis’ when costs outweigh benefits. (E) Two major independent evolutionary events of placental villi (reduced interdigitation) from a labyrinthine ancestor occurred in hoofed animals and primates (right column), which are born heavier and have longer gestation than their closest labyrinthine cousins (left column) [carnivores and (rodents + lagomorphs and tree shrews) respectively]. *P* value refers to the statistical comparison for combined gestation time and birth mass, for carnivores *versus* hoofed animals and rodents *versus* primates. Black dots in the primate plot show strepsirrhine primates.

Illustrative computational modelling of physiological processes within placental tissue structures can shed light on the underlying mechanisms of nutrient transfer efficiency (Faber, Thornburg & Binder, 1992; Schroder, 1995; Perazzolo, Lewis & Sengers, 2017; Laundon *et al*., 2023*a*, 2024*c*) (Fig. 4A,B). In Fig. 4A, we modelled transporter‐facilitated and passive diffusion in a section of mouse placenta (Cluster 1; hemochorial, labyrinthine) imaged by serial block face scanning electron microscopy (SBF‐SEM; see Appendix S1 for details). In transporter‐facilitated nutrient uptake (such as for amino acids), where transporters are localised in cell membranes, the number of cellular layers limits nutrient transfer in a stepwise fashion. For passive diffusion (such as for oxygen), transfer is determined by diffusion distance down a continuous gradient, whereby uptake will be reduced by greater distances. Fig. 4B shows how both transfer processes will be inhibited in placentas with increased tissue barriers between the placental interface and maternal circulation. Considered in terms of this single variable (i.e. the number of tissue layers separating fetal and maternal circulation), it is likely that physiological exchange is easier in Cluster 1 placentas.

However, the same intimate interdigitation and direct contact with maternal blood of Cluster 1 placentas that facilitates nutrient uptake also confers serious costs (Loke, 1982; Gluckman *et al*., 1992; Elliot, 2016) (Fig. 4C). If the costs of Cluster 1 placentation are indeed detrimental, then how was this form of placentation initially selected for and still retained in many groups of mammals today? We propose that for *r*‐selected small mammals with rapid gestation times, the spatiotemporal physiological demand is insufficiently high for these costs to materialise, as gestation is short. However, when clades of mammals expand into niches where large body mass and a longer gestation time are selectively advantageous, then the exponentially increased strain on the placenta rapidly causes the costs to exceed the benefits in terms of nutrient uptake and exposes the limitations of low‐investment placentation (Fig. 4D).

We term this a ‘placental crisis’, in an analogous way to Kuhn's (1962) description of a collapsing paradigm where an old framework no longer delivers its formerly consistent results. We can investigate this phenomenon using the mammal phylogeny as a ‘natural experiment’ (Fig. 4E). Villous placentation evolved from a labyrinthine ancestor (i.e. a reduction in interdigitation intimacy) in two major clades of mammals, hoofed animals and primates, with the former also being associated with an origin of epitheliochorial placentation (Wildman *et al*., 2006; Elliot & Crespi, 2009; Garratt *et al*., 2013; Carter, 2024). Comparing hoofed animals and primates with their closest labyrinthine cousins, carnivores and rodents (+lagomorphs and tree shrews) respectively, we can see that they have significantly higher gestation times and birth mass than their closest outgroups (Fig. 4E). This translates to hoofed animals and primates being larger and longer‐lived in adulthood, with gestation time correlated with lifespan (*P* < 0.001, *R* ^2^ = 0.60) and birth mass correlated with adult body mass (*P* < 0.001, *R* ^2^ = 0.95) in this data set as metrics of a slower life‐history strategy. Here, and below, when we refer to primates, we are specifically referring to haplorrhine primates (monkeys and great apes) with hemochorial discoid placentas and excluding strepsirrhine primates (lemurs and lorises) which have evolved epitheliochorial diffuse placentas (Fig. 2), making them functionally more similar to hoofed animals.

## IV. PLACENTAL CRISES: DISRUPTIVE SELECTION AND MATERNO‐FETAL CONFLICT DRIVE MAMMALIAN PLACENTAL EVOLUTION

**Fig. 5:**
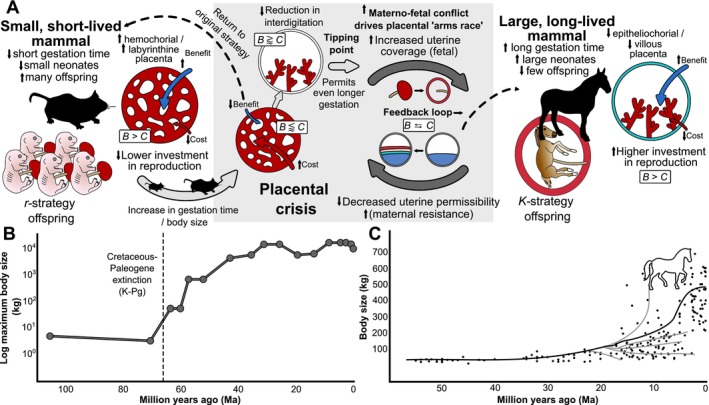
Placental structural diversification is triggered by a placental crisis and driven by disruptive selection and materno‐fetal conflict. (A) Visualisation of our integrated model proposing that maternal under‐investment triggers a placental crisis, where disruptive selection and materno‐fetal conflict interact to drive placental evolution and diversification. *B* = Benefit, *C* = Cost. (B) The fossil record shows that mammals were small bodied prior to the Cretaceous–Paleogene (K‐Pg) mass extinction event but increased exponentially in size afterwards. Plot adapted from Smith *et al*. (2010). (C) The evolution of large‐bodied mammals such as horses is indicative of rapid directional selection following a lag period. Dots are estimated body masses of extinct and extant equids. Black line shows the ancestry of extant horses, and grey lines show extinct lineages. Plot adapted from Nacarino‐Meneses (2023).

We present our integrated model outlining the mechanisms underpinning placental evolution in Fig. 5A. We note that different categories of placental types are under the control of either the mother or the fetus, although this is not often considered (Mossman, 1937; Samuel & Perry, 1972; Graham & Lala, 1991; Kshitiz *et al*., 2019; Laundon *et al*., 2024*c*). As the placenta is fetal in origin, uterine coverage and tissue interdigitation will be governed by the fetus. However, as the endometrium is maternal in origin, it is apparent that the number of cellular layers separating maternal blood from the placental tissue is controlled maternally (Samuel & Perry, 1972; Graham & Lala, 1991; Kshitiz *et al*., 2019; Wagner *et al*., 2022). Historically, this phenomenon was referred to as the ‘invasiveness’ of the placenta, implying that the fetus is the predominant driving agent, but this is not correct. Here we have opted for ‘uterine permissibility’ to flip the terminology better to reflect experimental evidence that the activity of maternal stromal cells permits or resists direct placental contact with maternal blood (Samuel & Perry, 1972; Graham & Lala, 1991; Kshitiz *et al*., 2019; Mika *et al*., 2022; Wagner *et al*., 2022). We argue that different placental features being controlled by either the fetus or mother is a key mechanism of placental diversification: an interactive ‘arms race’ between fetal and maternal interests. Although intergenomic conflict and materno‐fetal signalling have long been identified as a key aspect of placental evolution (Haig, 1993; Wang *et al*., 2013; Griffith & Wagner, 2017; Stadtmauer *et al*., 2025), this specific arms race has not been considered before.

A previous study identified multiscale structural adaptations to equid placental villi consistent with a response to reduced maternal uterine permissibility (Laundon *et al*., 2024*c*). The conflict between the mother's ability to withhold nutrients through decreased uterine permissibility, and the fetus’ interest in extracting increased nutrient resources by expanding placental uterine coverage or through multiscale structural innovations will result in an antagonistic arms race driving placental innovation through a positive feedback loop. Much like evolutionary arms races such as that between acacia thorns and the prehensile giraffe tongue (Zinn, Ward, & Kirkman, 2007; Dagg, 2014), this materno‐fetal arms race will also result in Red Queen dynamics (Van Valen, 2014) (a continuous evolutionary arms race between interacting parties), and a higher investment strategy from both parties.

Once a mammal enters placental crisis, selection pressures will initially push this clade back towards the initial highly *r*‐selected condition of short gestation times and very altricial young. However, if a fetus can offset its accumulated costs by evolving reduced interdigitation, such as through villous placentation, it can increase its body mass and gestation time deeper into the placental crisis at the expense of the mother's fitness. We refer to this condition as the ‘tipping point’ whereby the increased physiological strain on the mother will induce the materno‐fetal arms race between placental innovation and reduced uterine permissibility, causing a positive feedback loop which rapidly drives directional selection of large, precocial *K*‐selected offspring and high‐investment pregnancies. Selection against anything intermediate is evidence for disruptive selection driving two extremely *r*‐ or *K*‐selected fitness peaks (Wright, 1932) with a fitness valley in intermediate forms.

Our model is also consistent with the palaeontological understanding of mammal evolution. The ancestral hemochorial, labyrinthine, discoid placenta existed within the last common ancestor of placental mammals ~80–100 million years ago (Ma) (Álvarez‐Carretero *et al*., 2022; Foley *et al*., 2023), which would have been small‐bodied, like Cretaceous eutherian mammals from the fossil record (Ji *et al*., 2002; Orliac & O’Leary, 2016; Wang & Wang, 2023). Mammal body sizes remained low (relative to the upper body sizes of extant mammals) until the Cretaceous–Paleogene (K‐Pg) mass extinction event where maximum body sizes exploded exponentially (Alroy, 1999; Smith *et al*., 2010; Slater, 2014) (Fig. 5B). Thus, placental mammals experienced at least ~20 million years in an *r*‐shifted state, avoiding the manifestation of placental crises by low gestational demand. Niche expansion favouring larger *K*‐selected body sizes following the K‐Pg extinction of non‐avian dinosaurs would have initially had to enter a lag period of small‐bodied ancestors in reproductive crisis before exponential directional selection (through materno‐fetal arms races) in large‐bodied taxa, as observed in groups such as horses (Nacarino‐Meneses, 2023) (Fig. 5C) and cetaceans (Evans *et al*., 2012; Antar *et al*., 2023), but not in smaller groups like rodents (Evans *et al*., 2012).

## V. PRIMATES ARE IN CRISIS: MATERNAL UNDER‐INVESTMENT AS THE FOUNDATION OF HUMAN PLACENTAL DYSFUNCTION

**Fig. 6:**
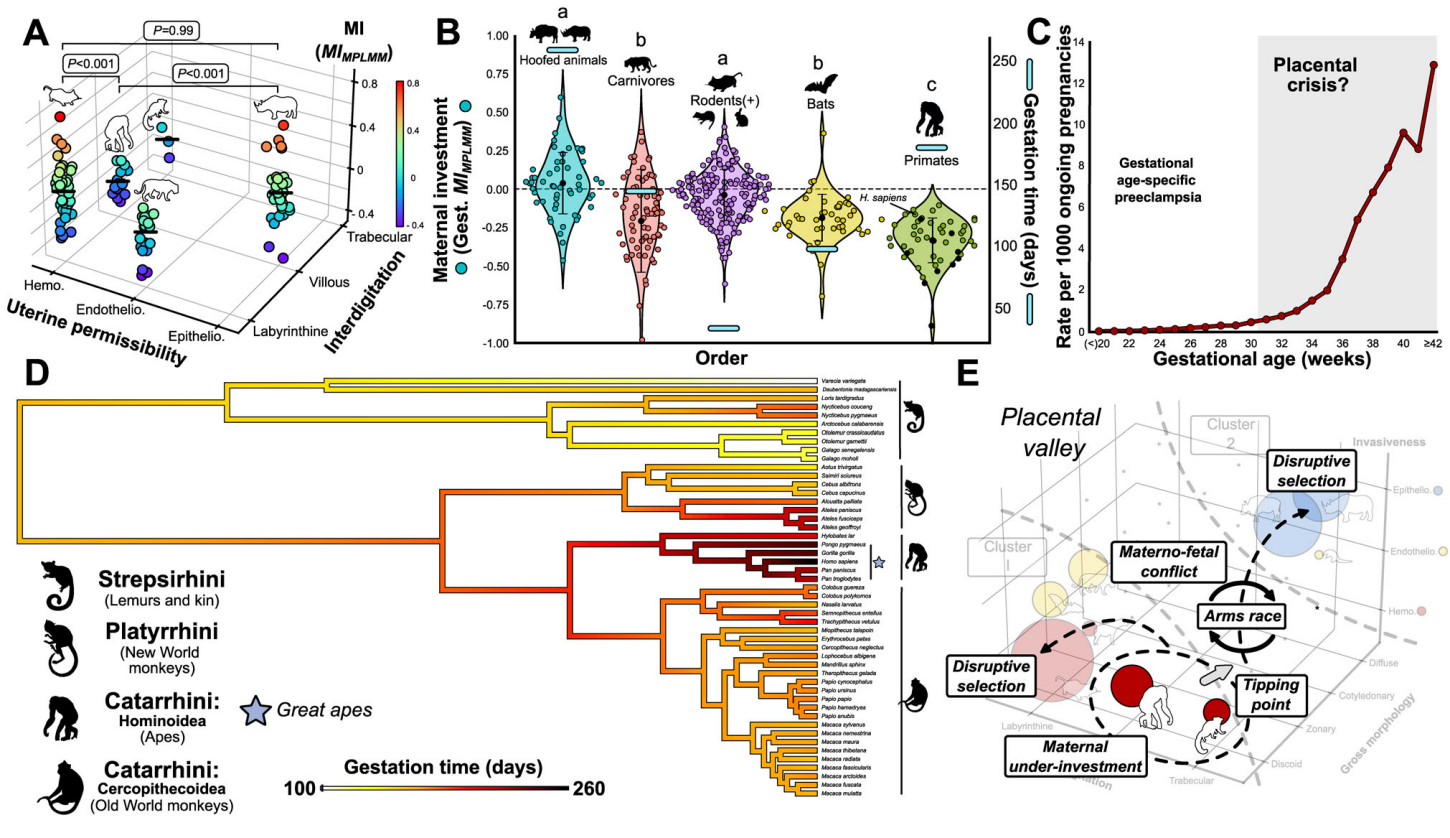
Maternal under‐investment and inappropriate placentation are the foundations of placental dysfunction in primates. (A) The differential maternal investment of mammalian placental types provides evidence for two adaptive peaks (hemochorial labyrinthine and epitheliochorial villous) fortified by disruptive selection, with maternal under‐investment for taxa in the ‘placental valley’. Silhouettes as in Fig. 3B. Black bars represent mean maternal investment. Epithelio., epitheliochorial; Endothelio., endotheliochorial; Hemo. hemochorial; MI, maternal investment; MI_MPLMM_, maternal investment from a multiple phylogenetic linear mixed model. (B) The relative gestational (Gest., left axis) maternal investment (dots and violins) of hoofed animals is significantly higher than any other group except for rodents. Maternal investment is shown with mean gestation time on right axis (cyan bars). Black dots in the primate plot show strepsirrhine primates. (C) Pre‐eclampsia is a gestational disorder which disproportionately manifests later in pregnancy. Plot adapted from Lisonkova & Joseph (2013). (D) Great apes, and specifically humans, have much longer gestation times than other primates. (E) Overlay of the mechanics of our model for maternal under‐investment and disruptive selection from Fig. 5A onto the distribution of placental types in extant mammals (Fig. 3B), proposing that primates are in a state of placental crisis.

If disruptive selection drives the segregation of two adaptive fitness peaks at opposite poles of the *r*–*K* continuum and placental morphospace, why do Cluster 1 pregnancies invest less than Cluster 2 pregnancies (Fig. 3D–F)? To understand this, we must examine these groups at higher resolution. Plotting the distribution of relative maternal investment by placental interdigitation and uterine permissiveness (Fig. 6A) highlights two adaptive peaks, with hemochorial labyrinthine mammals investing identical amounts into pregnancy as villous epitheliochorial mammals (*P* = 0.99). This may seem counter‐intuitive, given that the individual rodent‐type placentas are the lowest investment, but the MI_MPLMM_ metric is inclusive of litter size, which is large in rodents, so by operating within the fitness confines of the *r*‐selected peak this strategy reaches investment equality with the most expensive individual hoofed animal placentas. However, hemochorial villous mammals (including humans) invest significantly less than either peak (*P* < 0.001). Maternal investment only begins to decrease when a clade moves away from a disruptive fitness peak and down towards the placental valley. Indeed, the *r*–*K* continuum of reproductive strategies itself may be under disruptive selection in mammals in this way.

Examined taxonomically, hoofed animals and rodents invest significantly more in gestation than any other group, with primates investing significantly less than any other group (Fig. 6B) (*P* < 0.001). We suggest that this discrepancy underpins the gestational disorders of many primates not observed in other mammals and provides an explanation from evolutionary medicine. An example is pre‐eclampsia, a gestational disorder characterised by dangerously high maternal blood pressure that affects 3–5% of pregnancies in developed nations (Lisonkova & Joseph, 2013; Fisher, 2015), and whose incidence increases sharply with gestational age (Lisonkova & Joseph, 2013) (Fig. 6C). Other human gestational disorders that worsen over gestational age include gestational diabetes (Buchanan, Xiang & Page, 2012; McIntyre *et al*., 2019), placental accreta spectrum disorders (Jauniaux, Collins & Burton, 2018; Silver & Branch, 2018), and the vertical *in utero* transmission of pathogens (Arora *et al*., 2017; Megli & Coyne, 2022).

Pre‐eclampsia is a disease most associated with human pregnancies, but analogous symptoms have been observed in other great apes such as chimpanzees (*Pan troglodytes*), gorillas (*Gorilla gorilla*), and orangutans (*Pongo abelii*) (Carter, 2011; Crosley *et al*., 2013; Elliot, 2017; Walter *et al*., 2018). The evolutionary origins of pre‐eclampsia in this group have been argued to result from the deep trophoblast ‘invasion’ in great apes not seen in Old World monkeys, viewing this disorder through the efficiency paradigm as if the heightened nutrient transfer efficiency is excessive and pathological (Carter, 2011; Crosley *et al*., 2013). However, this would not explain case studies of pre‐eclampsia‐like symptoms in Old World monkeys like patas monkeys (*Erythrocebus patas*) (Palmer *et al*., 1979) and baboons (*Papio anubis*) (Cavanagh *et al*., 1974), nor their emergence in late gestation. Instead, we propose that the abundant placental dysfunctions of late gestation are a consequence of primates being in a state of placental crisis.

The hemochorial villous placenta and systemic maternal under‐investment is inappropriate for a clade of primate body sizes, longevity, and gestation length (Fig. 6D). Overlaying our mechanistic model for placental evolution onto the placental morphospace (Fig. 6E), we not only suggest that primates are in a placental crisis, but that the great apes have likely already reached the tipping point described in Fig. 5A given their villous placentation and high gestational demand. Given evolutionary time, and in the absence of medical interventions, chronic gestational dysfunction would induce increased maternal uterine resistance and trigger a materno‐fetal arms race, directionally selecting for large *K*‐selected primates with epitheliochorial villous placentas. Interestingly, maternal investment by strepsirrhine primates with epitheliochorial diffuse placentas (MI_MPLMM_ = 0.12) is significantly higher than in haplorrhine primates with hemochorial discoid placentas (MI_MPLMM_ = −0.16) (*P* = 0.001), suggesting this clade may have already escaped placental crisis by this route. Disruptive selection may also return the smallest bodied primates to labyrinthine placentation at the *r*‐selected peak. While the mechanistic causes of pre‐eclampsia are not known, the increasing understanding of resistance to implantation by the maternal decidua (Garrido‐Gómez *et al*., 2022) as a potential driving factor may even suggest an adaptive phenotype (at an evolutionary scale) in a subpopulation of primates whereby the mother is increasing uterine resistance to counteract excessive gestational demand.

For our hypothesis that some placentation strategies constitute lower investments to be fully validated, further empirical refinement of placental energetic budgets and tissue investment is required. We will need further to explore how intergroup variation in metabolism, such as torpor and hibernation in bats, influences placental energetics and investment. An immediate argument against our demonstration of under‐investment in primates would be that this metric does not account for the disproportionately large brain sizes of primates relative to hoofed animals, however, odontocete cetaceans (dolphins and some toothed whales) have relative brain sizes equal to or greater than most primates but, like the artiodactyls, have epitheliochorial villous placentas (Carter & Martin, 2012). Indeed, in our data set, odontocete cetaceans invest significantly more maternally (MI_MPLMM_ = 0.19) than haplorrhine primates (MI_MPLMM_ = −0.16) (*P* = 0.001). In addition, we will need to continue to refine the structural schema through which mammalian placentas are understood with three‐dimensional imaging and computational modelling (Laundon *et al*., 2023*a*,*b*, 2024*a*,*b*,*c*; Savatović *et al*., 2025).

Nevertheless, our inverted paradigm fits our current understanding of placental structural diversity, mammalian life‐history strategy, and evolutionary dynamics. We conclude that the ancestral mammalian placenta design was not a uniquely high‐efficiency innovation that permitted the placenta to dominate nutrient provisioning in the mammals. Indeed, none of the other 15 independent origins of vertebrate placentation converged on this strategy (Whittington *et al*., 2024). Rather, the ancestral mammalian placenta represents an idiosyncratic evolutionary accident resulting from the unique biology of this mammalian ancestor, such as the presence of platelets to mitigate against post‐partum haemorrhage (Martin & Wagner, 2019) and the capture of fusogenic retroviral proteins to allow trophoblast syncytialisation (Lavialle *et al*., 2013). Placentation became locked into mammalian biology through its tight intimacy with maternal physiology and the tens of millions of years placental mammals were trapped in small‐bodied *r*‐selected niches where the limitations of the ancestral placenta could not manifest. As such, we argue that the ancestral mammalian placenta may have hindered mammalian expansion into larger‐bodied niches rather than facilitated it.

## VI. CONCLUSIONS

(1) Vertebrates show high variation in reproductive strategy along the *r*–*K* continuum, but this does not mean that total reproductive investments are equal.
(2) Maternal investment metrics allow us to compare total maternal investment quantitatively among mammals, highlighting that some groups invest significantly more in reproduction than others.
(3) Placental structure is highly diverse across the mammals, but clustering of traits reveals tight co‐evolution of structures in a polar morphospace, indicative of disruptive selection.
(4) We propose that the relative efficiency of a placental configuration to transfer nutrients is not the explanatory paradigm of placental evolution, as previously suggested, but rather that differential maternal investment in reproduction is.
(5) We argue that the ancestral mammalian placenta is a product of low maternal investment in reproduction and that, as mammals expanded into larger bodied longer‐lived niches, this design triggered a ‘placental crisis’ characterised by chronic gestational dysfunction.
(6) Our model outlines how materno‐fetal conflict and disruptive selection interact during a placental crisis to drive mammalian diversification.
(7) Primates invest less in reproduction as a group than any other mammals, and we propose this is the foundation of primate‐specific gestational dysfunctions such as pre‐eclampsia.
(8) Taken together, we argue that maternal under‐investment and disruptive selection are the foundations of mammalian placental evolution and dysfunction.

## Supporting information


**Appendix S1.** Methodology.

**Data S1.** Full maternal investment and placental type data.

## Data Availability

The data sets for maternal investment metrics and placental types used for these analyses are available as Data S1.xls.
