# Supplementary material for: Placental crises: disruptive selection and maternal under‐investment as the foundations of mammalian placental evolution and dysfunction
Source: Biol Rev Camb Philos Soc. 2026 Jan 31;101(4):1627–40. doi: 10.1002/brv.70139 (PMC13326773; doi:10.1002/brv.70139)
Supplement: Supplementary file 1 — Appendix S1. Methodology. [file BRV-101-1627-s002.docx]

**Appendix S1. Methodology**

**(1) Placental structural analysis**

Our schema of placental structures (Fig. 2C–E) is based on our previous schema (Laundon *et al.*, 2023*a*), simplified according to methods in Carter (2024) to bin outliers into more functionally meaningful categories. Our data set of recorded placental structures (Fig. 3B) is from Elliot & Crespi (2009) (see Data S1), manually curated and refined using Carter (2024), and then plotted in 3D using matplotlib v3.8.0 (Hunter, 2007) run with Python 3.11.7 implemented in Jupyter Notebook 7.0.8.

**(2) Ancestral trait reconstruction**

The mammalian phylogenetic trees used for reconstructing ancestral placental traits (Fig. 2F–H), and the primate phylogeny in Fig. 6D, were produced from the mammalian phylogeny of Upham, Esselstyn & Jetz (2019) using the online resource <https://vertlife.org/data/mammals/>. Placental trait data were from Elliot & Crespi (2009) and gestation time data from Huijsmans *et al.* (2024) (see Data S1). A consensus tree was produced from 1,000 tree replicates using *consensus.edges* in Phytools (Revell, 2024) run with R v.4.4.0 implemented in RStudio v.2024.4.1.748. For reconstructing ancestral placental traits, the fit of different extended Mk (Markov k-state) model variations (Lewis, 2001) [ER = equal rates; SYM = symmetric rates; and ARD = all-rates-different, with both root priors = Fitzjohn and MCMC (Markov Chain Monte Carlo)] was compared by calculating Akaike Information Criterion (AIC) weights and scores for each variation. We simulated 1,000 stochastic character maps per structural category, weighting each model variation into the simulations calculated by AIC. Internal and ancestral node likelihoods were then calculated across the 1,000 maps by summary. For primates, the *contMap* function (Revell, 2013) was used to visualise gestation time against the consensus phylogeny.

**(3) Maternal investment metrics**

To visualise maternal investment from a multiple phylogenetic linear mixed model (*MI_MPLMM_*) between placental types (Fig. 3D-F), data were plotted as box plots with jittered points using the seaborn library (Waskom, 2021). Differences were compared statistically with either a *t*-test or Mann-Whitney-U test as appropriate following testing for normality and homogeneity with a Shapiro–Wilk and Levene’s test respectively, all in the SciPy v.1.11.4 library (Virtanen *et al.*, 2020). To recalculate *MI_MPLMM_* to incorporate only the gestational component of maternal investment (Fig. 3F), we reran the original R script from Huijsmans *et al.* (2024) (available at: <https://github.com/courtiol/mammalianMI>) substituting weaning mass with birth mass and investment duration with gestational time to remove the lactational portion of investment (see Data S1). Birth mass was not present in the original data set used by Huijsmans *et al.* (2024) and poorly sampled in the original amniote data set (Myhrvold *et al*., 2015) on which it was based, so we sourced these values from the PanTHERIA database (Jones, 2009). Gestation time and birth mass data for primates *versus* rodents and for hoofed animals *versus* carnivorans (Fig. 4E) were plotted and analysed from the original data set in Jupyter Notebooks as described above. Comparison of maternal investment metrics by invasiveness + interdigitation, and by taxonomic group (Fig. 6A, B), were likewise plotted in Jupyter Notebooks and analysed by either an ANOVA (Fig. 6A) or Kruskal-Wallis (Fig. 6B) test following normality and homogeneity assessment, and then by a Tukey’s or Dunn’s *post-hoc* test respectively in SciPy (Virtanen *et al.*, 2020).

**(4) Illustrative physiological modelling**

The labyrinthine zone of an E18.5 mouse (Fig. 4A) was imaged using serial block face scanning electron microscopy (SBF-SEM) using a ConnectomX Katana microtome inside a TESCAN CLARA SEM at 20×20×50 nm (Fig. 4A is a single slice). Sample processing and image segmentation followed Laundon *et al.* (2023*b*). Tissue was collected in accordance with local ethical approval from the University of Southampton (46381.A3). Image based simulations were performed in COMSOL Multiphysics (v6.3; COMSOL AB, Stockholm, Sweden) as in Lewis *et al.* (2022). Parameters for glucose were used to simulate membrane transport and diffusion. Transport at the interface between each tissue layer and the maternal and fetal blood was modelled using a carrier-based transporter model for GLUT 1 (glucose transporter Type 1) (Barros *et al.*, 2007). The maximum uptake rate was estimated from Jansson, Wennergren & Powell (1999) and the same transport parameters were applied to each interface. The boundary conditions for the normalised maternal and fetal blood concentrations were set to 1 and 0 respectively. The intracellular diffusion coefficient was adopted from Kreft *et al.* (2013), scaled to 37 °C.

**(5) Other plots and statistical outputs**

Figs 1B (Abbot & Capra, 2017), 5B (Smith *et al.*, 2010), 5C (Nacarino-Meneses, 2023), and 6C (Lisonkova & Joseph, 2013) were reproduced directly from their original sources as cited and only edited aesthetically. The maternal investment between Cluster 1 and Cluster 2 placentas in Fig. 3D was compared by *t*-test (*t* = –2.5, *P* = 0.014). The maternal investment between hoofed animals and all other boreoeutherians in Fig. 3E was compared by a Mann-Whitney U test (*U* = 25,974, *P* < 0.001). The comparison of gestational investment only between the two groups in Fig. 3F was tested by Mann-Whitney U test (*U* = 14,189, *P* < 0.001). The correlation between gestation time and lifespan (*r* = 0.78, *P* < 0.001, *R*^2^ = 0.60) and birth mass and adult body mass (*r* = 0.97, *P* < 0.001, *R*^2^ = 0.95) in our data set were both compared using a Pearson’s test. The maternal investment between placental types shown in Fig. 6A was compared by ANOVA (*F* = 8.39), with a Tukey’s *post-hoc* test. Comparison of investment between mammal groups was tested with a Kruskal-Wallis test (*H* = 117.9, *P* < 0.001) and Dunn’s *post-hoc* test. Comparison between maternal investment in strepsirrhine and haplorrhine primates was carried out using a Mann-Whitney U test (*U* = 115.0, *P* = 0.001), and between odontocetes and primates also with a Mann-Whitney U test (*U* = 509.0, *P* = 0.001).
